# A novel one-day phage-based test for rapid detection and enumeration of viable *Mycobacterium avium* subsp. *paratuberculosis* in cows’ milk

**DOI:** 10.1007/s00253-020-10909-0

**Published:** 2020-09-24

**Authors:** Antonio C. G. Foddai, Irene R. Grant

**Affiliations:** Institute for Global Food Security, School of Biological Sciences, Queen’s University Belfast, Belfast, Northern Ireland BT9 5DL UK

**Keywords:** Milk, *Mycobacterium avium* subsp. *paratuberculosis* (MAP), Phagomagnetic separation, Quantitative PCR (qPCR), Viability assay

## Abstract

**Abstract:**

Bacteriophage-based methods for the rapid detection of viable *Mycobacterium avium* subsp. *paratuberculosis* (MAP) in veterinary specimens are a recent addition to the Johne’s disease diagnostic toolbox. Here, we report the use of D29 mycobacteriophage-coated tosylactivated paramagnetic beads to capture and concentrate MAP cells from samples (termed phagomagnetic separation, PhMS) and then naturally lyse viable MAP cells (from the inside out) to provide DNA for IS900 qPCR purposes. Transmission electron microscopy confirmed that D29 phages had bound to beads in the correct orientation and that the phage-coated beads captured MAP cells from a suspension. During test optimization, conventional IS900 PCR results were used to subjectively assess the effect of different phage:bead coating ratios, differing amounts of coated beads during PhMS, optimal incubation time post-PhMS to obtain maximal MAP DNA, and the potential benefit of a brief heat shock (55 °C/1 min) prior to IS900 TaqMan qPCR. The limit of detection 50% (LOD_50%_) of the optimised PhMS-qPCR assay was 10.00 MAP cells/50 ml milk (95% CI 1.20–82.83). Finally, in order to demonstrate the new assay’s ability to detect viable MAP in naturally contaminated milk, bulk tank milk samples from 100 dairy farms were tested. Forty-nine (49%) of these tested PhMS-qPCR-positive, with viable MAP numbers detected ranging from 3–126 MAP/50 ml. The novel PhMS-qPCR assay is a sensitive, specific and easy-to-apply phage-based assay for viable MAP, with potential application for milk surveillance or diagnosis of Johne’s disease.

**Key points:**

*• Phage-coated magnetic beads could capture, concentrate and lyse MAP cells from milk.*

*• PhMS-qPCR assay proved to be a rapid, sensitive and specific test for viable MAP.*

*• A potential application of PhMS-qPCR assay for milk surveillance was demonstrated.*

## Introduction

Spread of Johne’s disease (JD) is becoming a big economic problem for dairy farmers worldwide (European Food Safety Authority 2017). JD (also known as Paratuberculosis) is a chronic intestinal infection caused by *Mycobacterium avium* subsp. *paratuberculosis* (MAP) which leads to chronic diarrhea, weight loss and declining milk production. Control of JD is currently very difficult due to the lack of sensitive tests able to detect early stages of infection. Animals generally become infected with MAP at a young age but can remain symptomless for several years. During this, time MAP is being shed in faeces and milk, leading to disease transmission within herds. As bacterial shedding precedes an antibody response against MAP, existing humoral tests can only detect animals in advanced stages of JD (Beaver et al. 2017; Van Schaik et al. 2003). MAP can be cultured from faeces, milk and blood of animals (Bower et al. 2010; Gilardoni et al. 2012), but because of the long doubling time of MAP, the method is slow and takes weeks to yield results. Detection of MAP DNA from environmental or veterinary samples is also complicated. Molecular tests such as PCR and real-time quantitative PCR theoretically offer a faster and specific detection of MAP DNA, but their detection capability largely depends on the quality of tested samples. DNA extracted from clinical specimens is generally a co-purified sample containing high amounts of non-mycobacterial DNA, proteins and other potential PCR inhibitors that might have an adverse effect on final test results (Christopher-Henning et al. 2003; Collins et al. 2006; Radomsky et al. 2013).

Over recent years, there has been increasing interest in bacteriophage-based methods as a potential alternative to culture for the rapid detection and enumeration of viable MAP. A phage-based assay for detecting MAP was first reported by Stanley et al. (2007), who demonstrated that the FASTPlaqueTB phage amplification assay for detecting *Mycobacterium tuberculosis* in sputum (commercially available at the time from Biotec Laboratories, Ipswich, UK) could be repurposed for detection of viable MAP in cows’ milk. Foddai et al. (2009) subsequently optimised the FASTPlaqueTB assay conditions to ensure accurate quantitation of the number of viable MAP present in milk. The main changes introduced were a longer incubation time after phage infection (3.5 h instead of 1 h) before plating with *Mycobacterium smegmatis* and molten 7H9 agar, and virucide (ferrous ammonium sulphate, FAS) treatment at the 2-h point within this incubation period rather than at 1 h just before plating. The ability of the optimised phage assay as a tool to monitor the inactivation kinetics of MAP in milk during heat treatment was subsequently demonstrated (Foddai et al. 2010a). Later, the same researchers combined the optimised phage amplification assay with peptide-mediated magnetic separation (PMS) employing MyOne Tosylactivated Dynabeads coated with two biotinylated peptides aMp3 and aMptD, originally described by Stratmann et al. (2002, 2006). The PMS-phage assay was optimised in order to maximize sensitivity for MAP detection (Foddai et al. 2010b) and then used to detect viable MAP in bovine milk and faeces (Foddai et al. 2011). PMS achieves two important things in advance of the phage amplification assay: (1) selective capture of mycobacterial cells from other microorganisms present, and (2) physical separation of MAP cells from the complex milk sample matrix. This means that the D29 phages can encounter the MAP cells in a sample more easily, since milk components are largely eliminated. There is also the potential to 10-fold concentrate MAP cells from a milk sample if the beads are resuspended in a smaller volume (0.1 ml rather than 1 ml) of broth after PMS.

At Queen’s University Belfast (QUB), we have been using the PMS-phage assay with only a few minor tweaks, in terms of milk sample preparation mainly (Foddai and Grant 2015), for many years now, chiefly to test for viable MAP in milk (Foddai and Grant 2017; O’Brien et al. 2018) and calf milk replacer (Grant et al. 2017). Our studies have consistently shown that the PMS-phage assay is a very sensitive test for detecting viable MAP, and a promising rapid alternative to MAP culture, which takes a long time to return results but is still considered the gold standard method for demonstrating the presence of viable MAP in veterinary samples. In our hands, the PMS-phage assay performs consistently well since we have become proficient in its application and recognise the key steps within the assay that need to be performed correctly to avoid false positive or false negative results. However, when researchers in other laboratories have attempted to adopt the optimised phage assay or the PMS-phage assay, technology transfer has generally not been a smooth process (e.g. Butot et al. 2019), and considerable training and troubleshooting has been needed from QUB researchers. We acknowledged some time ago (Foddai and Grant 2017) that the PMS-phage assay has a complex, multi-step protocol that does not lend itself well to high-throughput testing of milk samples. There are a couple of key parts of the PMS-phage assay protocol that must be performed with care; otherwise, false positive plaques due to non-inactivated seed phages (as a consequence of ineffective virucide treatment) or release of progeny phages before plating in agar (due to non-adherence to stipulated incubation times) may result. False negative results may also occur because plaque PCR does not confirm the presence of MAP DNA within the maximum 10 plaques harvested (irrespective of the number of plaques present).

If phage-based assays for viable MAP are to have any future application for the diagnosis of JD, then a much simpler, user-friendly test protocol is going to be required. This urgent need prompted us to develop a novel 1-day phage-based test for viable MAP by employing the D29 mycobacteriophages in a different manner—for physical capture and natural lysis of MAP cells in advance of MAP-specific qPCR, rather than phage amplification within MAP cells and a plaque assay endpoint. The objectives of this study were to (1) successfully coat D29 phages onto paramagnetic beads and use these phage-coated beads for phagomagnetic separation (PhMS) of MAP cells from milk; (2) determine the best protocol for harvesting DNA released from viable MAP cells lysed by action of the D29 phages; and (3) combine the PhMS and DNA harvesting steps with quantitative IS900 PCR (qPCR) to produce a rapid, sensitive and specific PhMS-qPCR assay for viable MAP in milk. From the outset, we acknowledge the existence of the Actiphage® Rapid test, which is a commercially available rapid phage-based test from PBD Biotech Limited (Thurston, UK) based on technology developed by Drs. Cath Rees and Ben Swift at the University of Nottingham, UK (Swift et al. 2019). The PhMS-qPCR test we describe here may appear similar to the Actiphage® Rapid test, but it has a different *modus operandi*.

## Materials and methods

### Bacterial strains and growth conditions

Three MAP strains, including one reference strain ATCC19698, one bovine isolate B4 and the strain 796PSS originally isolated from retailed pasteurized milk (Grant et al. 2002), were used in this study. All MAP strains were grown to stationary phase in a static incubator for 2–3 weeks at 37 °C in screw cap glass vials (Cole-Parmer, UK) using 5 ml modified Middlebrook 7H9 broth (Pozzato et al. 2011), containing 0.47% 7H9 powder (Difco), 0.1% Casitone and 0.5% glycerol (both from Sigma), 10% (v/v) Oleic Albumin Dextrose Catalase (OADC) supplement (Difco) and 2 μg/ml mycobactin J (Synbiotics Europe SAS, Lyon, France). *Mycobacterium smegmatis* mc^2^ 155, to be used for the plaque assay, was cultivated at 37 °C to stationary phase for 3 days in conventional Middlebrook 7H9 medium enriched with 10% (v/v) OADC supplement (Difco) without the addition of mycobactin J.

### Preparation of MAP inoculum

Bacterial suspensions used to spike broths and milk samples tested in this study were prepared as previously described by Foddai and Grant (2015). Briefly, glass vials containing stationary MAP broth cultures were processed through ultrasonication, applied at 37 kHz for 4 min on ice in an Ultrasonic PH 30 (Fisher Scientific Ltd., Loughborough, UK) in order to disperse clumps of mycobacteria. The purity of de-clumped MAP suspensions was then verified by Ziehl-Neelsen (ZN) staining in order to ensure presence of only red acid-fast cells. The number of MAP cells per ml of broth was estimated by measuring the optical density at 600 nm (OD_600_) using a WPA CO8000 cell density meter (SISLAB, Cornaredo, Italy). For each sample, optical density was adjusted to OD_600_ 0·1 (approximately 10^6^–10^7^ MAP cells per ml) followed by serial dilution of cultures in phosphate-buffered saline (PBS) containing 0.05% (v/v) Tween 20 (PBS–TW20, Sigma). Four spiking levels (10^4^–10^3^, 10^3^–10^2^, 10^2^–10, and approximately 10 MAP per ml) were finally used to prepare the artificially contaminated broth and milk samples tested in this study to assess recovery rates of MAP by phagomagnetic separation and the new phagomagnetic separation (PhMS)-qPCR test.

### Propagation of D29 mycobacteriophage

D29 mycobacteriophage (originally gifted to Prof. Irene Grant by Dr. Ruth McNerney, London School of Hygiene and Tropical Medicine circa 2008) was propagated in agar plates containing *M. smegmatis* mc^2^ 155 (also originally received from Dr. Ruth McNerney). Five or six Middlebrook 7H9 agar plates containing around 200 to 300 plaques were flooded with 5 ml 7H9 broth supplemented with 10% OADC and 2 mM CaCl_2_ then incubated overnight at 37 °C, followed by another overnight incubation at 4 °C. The broth containing phage particles was recovered and centrifuged at 2,500×*g* for 10 min, the supernatant was filtered through 0.22-μm Millex GP Millipore Express PES membrane filter units (Millipore UK Limited, Croxley Green, UK). The number of D29 phage particles present in this stock culture was determined by titration, which involved serial dilution of the phage stock in 7H9 broth and plating in Petri dishes along with 1 ml *M. smegmatis* mc^2^ 155 culture and tempered (55 °C) molten 7H9 agar. The D29 stock solution used to prepared phage-coated paramagnetic beads was standardized to a concentration of 10^11^ PFU/ml and then stored at 4 °C until required.

### Optimization of protocol to prepare D29 phage-coated beads

BcMag^TM^ Tosylactivated 1-μm paramagnetic beads (Bioclone Inc., San Diego, USA) were coated with D29 phages via covalent linking with amino groups on the surface of mycobacteriophage. Phage-coated paramagnetic beads were prepared using a combination of the manufacturer’s instructions and the bead coating protocol described by Laube et al. (2014) for coating paramagnetic beads with *Salmonella*-specific phages. Briefly, upon arrival in the laboratory the BcMag Tosylactivated beads (150 mg) were suspended in 1.5 ml of isopropanol (Sigma), to give a stock bead concentration of 100 mg/ml, and stored at 4 °C as recommended by the manufacturer. A portion (10 mg) of resuspended paramagnetic beads (approximately 1.7 × 10^9^ particles) was then washed three times with 1 ml 0.1 M sodium carbonate/bicarbonate buffer pH 9.5, and covalently coated to D29 bacteriophages (10^10^ PFU/ml) previously resuspended in 1 ml of the same buffer. Coating between paramagnetic beads and phage particles proceeded overnight (~ 12 h) at 37 °C with continuous mixing (30–40 rpm) on a Stuart rotator mixer (Cole-Parmer, Stone, UK). After coating, beads were captured on a magnetic rack and the supernatant was recovered and tested by the phage plaque assay to permit assessment of the efficiency of coupling by comparing PFU counts before and after coating. Impact of blocking paramagnetic beads after coating with phages with BSA was also assessed by splitting coated beads into two portions. One portion of coated beads was resuspended in PBS pH 7.4. The other portion was incubated overnight with 1 ml PBS containing 0.5% BSA, magnetically captured and finally resuspended in 1 ml PBS containing 0.2% BSA. Capture ability of phage-coated beads (10 μl) was assessed by magnetic separation (MS) carried out using the Dynal BeadRetriever (Life Technologies, Paisley, UK) and testing broth samples spiked at different levels with MAP. After MS, the quantity of recovered MAP cells was subjectively assessed by conventional IS900 PCR (Millar et al. 1996, see details below) applied on DNA extracted from samples tested before and after MS by boiling at 99 °C for 25 min and centrifugation to clarify supernatant. Optimization of the amounts of phage-coated paramagnetic beads necessary to achieve the desired method sensitivity was also assessed. MS using decreasing amounts of D29 phage-coated paramagnetic beads (10 μl, 5 μl, 1 μl) was carried out on 1 ml broth samples spiked at four different levels with MAP; estimated amounts of paramagnetic beads were 1.7 × 10^7^, 8.5 × 10^6^ and 1.7 × 10^6^ beads/ml of test sample, respectively. After each MS, recovery of bacteria was subjectively assessed by conventional IS900 PCR. To conclude optimization, capture ability of magnetic beads (10 mg, approximately 1.7 × 10^9^ beads) prepared with decreasing amounts of D29 phage particles was assessed. Phage-coated beads with differing phage/magnetic bead (MB) ratios (10, 5, 1, 0.1 MAP PFU/MB) were prepared and used for MS. For each type of coated bead, efficiency of coupling was assessed by phage plaque assay test by comparing MAP PFU counts before and after coating, and recovery of MAP cells was subjectively assessed by conventional IS900 PCR.

### Visualisation of the immobilized phage particles on paramagnetic particles by transmission electron microscopy

In order to confirm that D29 phage particles had been immobilized onto the paramagnetic beads in the correct orientation for MAP capture (i.e. tail outwards), as well as demonstrate the successful capture of MAP cells after PhMS using D29 phage-coated beads, transmission electron microscopy (TEM) was carried out. For the TEM, 10 μl of the D29 phage-coated beads were washed three times in 1 ml molecular grade water (Sigma) before being diluted 1:100 in the same medium containing 2% glutaraldehyde. A small quantity of resuspended beads (10–20 μl) was coated overnight onto carbon-coated EM grids (TAAB Laboratories Equipment Limited, Aldermaston, UK) at room temperature in a sealed dark box. The grids were then stained for 20 min with 2% uranyl acetate solution (TAAB Laboratories Equipment Limited) previously filtered through 0.2-μm syringe filter to remove precipitate. Excess of stain was soaked up using a Whatman filter paper and grids were finally visualized using a JEOL JEM-1400 Plus Transmission Electron Microscope (JEOL UK, Welwyn Garden City, UK) operated at 100 kV. Images were recorded using a JEOL Ruby 8MP Bottom mounted CCD digital Camera. A similar protocol was applied to prepare post-PhMS samples for TEM, in order to visualize successful capture of MAP cells by D29 phage-coated beads. Gamma-irradiated MAP broth suspensions containing 10^4^–10^5^ MAP cells were employed for the TEM work for health and safety reasons. After, PhMS samples were resuspended in molecular grade water containing 2% glutaraldehyde and processed as for D29 phage-coated beads alone.

### Recovery of MAP cells by D29 phage-coated paramagnetic beads from spiked milk

Once optimal conditions for coating beads with D29 phages had been established, recovery rates of the optimal D29 phage-coated beads were assessed through testing four replicates of 50 ml UHT milk (purchased from a local supermarket) spiked with decreasing amounts of MAP cells (from 10 to 10^4^ MAP/50 ml). Before being subjected to MS, each artificially contaminated milk sample was centrifuged at 2,500×*g* for 15 min, cream and whey fraction were discarded and the milk pellet, which contains the vast majority of MAP bacterial load (Foddai and Grant 2015), was resuspended in 1 ml of PBS-TW20. Three different amounts of D29 phage-coated beads (10 μl, 15 μl and 20 μl/ml of resuspended milk pellet) were tested. PMS using Dynabeads MyOne Tosylactivated beads (Life Technologies) coated with two biotinylated peptides, aMp3 and aMptD (Foddai et al. 2010), was applied in parallel as a control, in order to assess which one of the three test conditions achieved similar analytical sensitivity to the existing PMS test.

### Optimization of post-PhMS conditions before qPCR

This experiment was carried out in order to identify optimal post-PhMS conditions to maximize quantity of DNA released from viable MAP cells infected and then lysed by the action of the D29 phages. Broth suspensions containing approximately 10^4^ MAP cells/ml were processed through PhMS using D29 phage-coated BcMag paramagnetic beads. Following PhMS, bead samples were resuspended in 50 μl of 7H9 Middlebrook broth containing 10% OADC and 2 mM CaCl_2_ and incubated, without shaking, for 1, 2, 3 and 4 h at 37 °C. At each incubation time, samples were centrifuged at 10,000×*g* for 1 min and a small portion (10 μl) of the sample supernatant was processed through conventional IS900 PCR to check for the presence of DNA released from D29-infected MAP cells. In order to maximize the quantity of DNA suitable for PCR purposes, and potentially reduce the time of the test, the impact of introducing a brief, mild heat shock treatment at 55 °C for 1 min applied after each incubation time was evaluated. This mimicked the brief heat shock experienced when a phage-infected sample is plated with molten agar at 55 °C during the original phage amplification assay (Foddai et al. 2009). Quantity of DNA released from viable MAP cells infected with D29 bacteriophages was then subjectively assessed based on intensity of PCR bands achieved from heat-shocked and unheated samples. The same experiment was subsequently carried out on 1 ml broth suspensions containing approximately 10^4^ MAP cells previously subjected to a 10 kGy dose of γ-radiation to completely inactivate them, in order to verify that non-viable MAP cells did not yield any PCR products after PhMS.

### Conventional IS900 PCR

Conventional IS900 PCR during the first part of this study to optimise coating protocol for D29 phage-coated beads was applied as previously described by Millar et al. (1996) with some modifications. Each PCR reaction was carried out in a final volume of 50 μl containing 1× Platinum™ Green Hot Start PCR Master Mix, 1 U Platinum™ Taq Green Hot Start DNA polymerase, 200 μM of each dNTP (all Thermo Fisher Scientific, Paisley, UK), 3 mM MgCl_2_, 2 μM forward primer P90 (5′ GAA GGG TGT TCG GGG CCG TCG GCC TTA GG 3′), 2 μM reverse primer P91 (5′ GGC GTT GAG GTC GAT CGC CCA CGT GAC) and 10 μl genomic DNA extracted by heating pre- and post-MS samples at 99 °C for 25 min. PCR was carried out on a Techne™ Prime thermal cycler (Cole-Parmer), with the following conditions: 4 min of initial denaturation at 95 °C, 37 cycles of 95 °C for 30 s, 59.5 °C for 30 s and 72 °C for 30 s, followed by a final elongation at 72 °C for 4 min. PCR products were visualized by agarose gel electrophoresis. The expected size of the IS900 PCR band was 394 bp.

### Evaluation of three real-time quantitative qPCR protocols combined with new PhMS method

Three different qPCR protocols were evaluated as potential end-point detection methods to be combined with the novel D29-based PhMS method: an IS900 SYBR Green qPCR (Bull et al. 2014), an IS900 TaqMan qPCR (Sidoti et al. 2011) and the commercially available Techne^TM^ PrimePRO qPCR DNA detection Kit, *Mycobacterium avium* subspecies *paratuberculosis* (Techne Ltd., product code TKIT08017M) targeting the f57 gene. IS900 SYBR Green qPCR was performed as previously described by Bull et al. (2014), with minor adjustments. Briefly, each reaction was carried out on a final volume of 20 μl and containing 10 μl 2× SYBR green Master Mix (SensiFAST™ SYBR® Hi-ROX Kit, Bioline Reagents Limited, London, UK), 1 μM forward primer (AV1: ATGTGGTTGCTGTGTTGGATGG), 1 μM reverse primer (AV2: CCGCCGCAATCAACTCCAG) and 2 μl template DNA. PCR conditions were 95 °C for 10 min, followed by 40 cycles of 95 °C for 30 s, 58 °C for 1 min and 72 °C for 1 min. Standard melt curve analysis was applied at the end to evaluate the specificity of each qPCR positive reaction, consisting of 10 s at 95 °C followed by a 0.2 °C/s temperature increment between 55 and 95 °C. IS900 TaqMan qPCR was carried out as previously reported by Sidoti et al. (2011) with some modifications. Each reaction was carried out on a final volume of 20 μl and included 10 μl 2× TaqMan qPCR Master Mix (SensiFAST™ Probe® Hi-ROX Kit, Bioline Reagents Limited), 1.5 μM forward primer IS900QF CCGGTAAGGCCGACCATTA, 1.5 μM reverse primer IS900QR ACCCGCTGCGAGAGCA, 6 pmoles IS900 TaqMan Probe FAM-CATGGTTATTAACGACGACGCGCAGC-TAMRA and 4 μl template DNA. PCR cycling conditions included an initial warm up section of 50 °C for 2 min, a denaturation step at 95 °C for 10 min followed by 40 cycles of 95 °C for 15 s and 60 °C for 1 min. The commercially available Techne^TM^ PrimePRO qPCR DNA detection Kit (Techne^TM^) gene was used following the manufacturer’s instructions. Briefly, each qPCR reaction (20 microliters) included 10 μl 2× qPCR Master Mix, 1 μl MAP primer/probe mix, 1 μl internal endogenous control primer/probe mix, 3 μl RNAse/DNAse-free water and 5 μl template DNA. PCR cycling conditions involved an initial 37 °C for 10 min, denaturation at 95 °C for 2 min, followed by 50 cycles of 95 °C for 10 s and 60 °C for 1 min. All qPCR reactions were carried out using an Eco™ Real-Time PCR system (Illumina, Inc., San Diego, USA) and the associated software. A standard curve was included in each qPCR run, to permit quantitation of MAP detected. Standard curve consisted of DNA samples extracted from a serial dilution of broth suspensions containing 10 to 10^4^ MAP cells/ml.

### Recovery of MAP cells from artificially contaminated milk samples

Analytical sensitivity of the new PhMS-qPCR was firstly evaluated by testing 50 ml UHT milk samples spiked at four levels (10^3^–10^4^, 10^2^–10^3^, 10–10^2^, 1–10) with MAP. A preliminary set of experiments was carried out with three MAP strains, ATCC 19698, B4 and 796PSS tested individually. In order to better estimate the limit of detection (LOD_50_) of the new test, a blind trial to test both spiked and non-spiked UHT milk samples was arranged. Twenty-five 50 ml UHT milk samples, including 20 samples spiked at four levels of MAP contamination (five at 10^3^–10^4^, five at 10^2^–10^3^, five at 10–10^2^, five at 1–10) and five non-spiked, were included in the trial. The operator was blinded to sample details until test results became available. Test conditions used for this spiked milk analysis were PhMS using D29 phage-coated beads, post-PhMS incubation of samples for 2 h at 37 °C, brief heat shock at 55 °C for 1 min, brief centrifugation at 10,000×*g* for 1 min to sediment cell debris and IS900 TaqMan qPCR applied on the DNA released from MAP cells at the end of lytic cycle.

### Shelf-life of D29 phage-coated beads

The shelf-life of D29-coated BcMag^TM^ paramagnetic beads stored at 4 °C was determined by evaluating the impact of storage for up to 1 year on MAP cell capture. Along with PBS pH 7.4, the resuspension buffer used for the first stock of D29-coated BcMag^TM^ prepared in this study, three other storage buffers (50 mM Tris HCl pH 9, 50% glycerol and 7H9-OADC-2 mM CalCl_2_ broth), were also evaluated. Four stocks of D29-coated beads resuspended in four preservation buffers were prepared and stored for up to 6 months at 4 °C with testing at monthly intervals by PhMS and conventional IS900 PCR. The MAP capture capability of each stock of phage-coated beads was assessed over time by testing broth suspensions spiked at different levels (10 to 10^4^ cells/ml) with MAP.

Maintenance of infectivity of the phage particles coated onto the paramagnetic beads was also monitored over time, to complete the shelf-life assessment of the D29-coated beads. A stock of D29 phage-coated beads stored for up to 12 months after preparation was tested monthly via plaque assay to check impact of prolonged refrigeration on D29 activity. Survival of D29 phages coated onto paramagnetic beads was estimated based on plaque-forming units (PFU/ml) generated by dilutions of coated beads being plated with *M. smegmatis* mc^2^ 155 and molten 7H9 agar at monthly intervals during refrigerated storage.

### Application of the novel PhMS-qPCR for testing bulk tank milk samples

Between September and October 2019, samples of bulk tank milk from 100 Northern Ireland dairy farms collected for milk testing purposes by Dale Farm Ltd. (Belfast, Northern Ireland) were provided to QUB for MAP testing by the novel PhMS-qPCR assay. Upon arrival at the QUB laboratory, milk samples (generally ~ 30–40 ml) were tested immediately for viable MAP by the PhMS-qPCR assay. To aid release of MAP cells for capture, and also minimise size of cream fraction, milk samples were pre-warmed at 37 °C for 10 min in a water bath before centrifugation at 2,500×*g* for 15 min. After centrifugation, the cream and whey fractions were discarded, and the pellet fraction was resuspended in 1 ml of PBS-TW20 and tested for MAP. After PhMS, bead samples were incubated at 37 °C for 4 h (rather than the 2 h used for laboratory-grown MAP) before mild heat shock treatment and subsequent IS900 qPCR. Based on some preliminary observations, this 4-h incubation time was found to be more appropriate for MAP in naturally infected milk samples, particularly when testing samples previously subjected to prolonged refrigeration before delivery. The quantity of MAP detected by qPCR per 30–40 ml milk volumes was corrected to quantity of MAP per 50 ml milk (the more usual manner in which MAP counts in milk are expressed), to permit comparisons between results for the variable volumes of BTM tested.

### Statistical analysis of results

The statistical significance of differences between mean plaque counts observed (a) before and after coating of beads and (b) before and after prolonged storage at refrigeration temperature during the shelf-life experiments was assessed by a paired *t* test using GraphPad InStat software (GraphPad Inc., San Diego, USA); results were statistically significant when *P* value was < 0.05. LOD_50_ and associated 95% confidence limits of new PhMS-qPCR assay was estimated using the generalized Spearman-Karber LOD_50_ calculation for four-level spiking protocols (AOAC International 2006).

## Results

### Optimization of protocol for preparation of D29 phage-coated beads

Covalent immobilization of D29 phage particles onto paramagnetic beads was assessed by coupling phage particles (approximately 10^10^ PFU/ml) to 10 mg of BcMag^TM^ Tosylactivated beads and then evaluating the phage concentration before and after the immobilization step. PFU counts repeatedly showed significant 3 log_10_ PFU/ml decreases (*P* value = 0.00066, paired *t* test) after coating, thus demonstrating successful immobilization of phage particles onto the paramagnetic beads by adopting the Laube et al. (2014) protocol. Mean coupling efficiency, obtained by comparing PFU counts before and after coupling for phage-coated paramagnetic beads, was 99.93 ± 0.02 %.

**Fig. 1:**
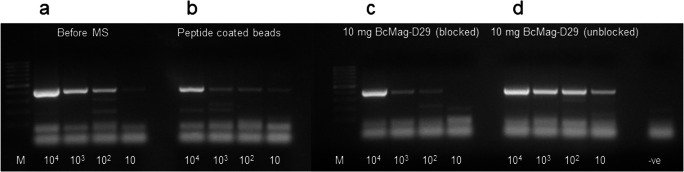
Comparison of MAP cell capture from spiked broth suspensions (10 to 10^4^ cells/ml) by magnetic separation using peptide- and phage-coated paramagnetic beads assessed by IS900 PCR. Results are for DNA extracted by boiling at 99 °C for 25 min from samples **a** before magnetic separation (before MS) and **b** after peptide-mediated MS involving a 50:50 mixture of MyOne Tosylactivated beads (Life Sciences) covalently coated to two biotinylated peptides aMp3 and aMptD (PMMS). **c** D29 phage-coated BcMag Tosylactivated beads blocked overnight with PBS-0.5% bovine serum albumin, and **d** unblocked D29 phage-coated BcMag Tosylactivated beads. M: TrackIt 100 bp DNA Ladder (Thermo Fisher Scientific), -ve: negative control

**Fig. 2:**
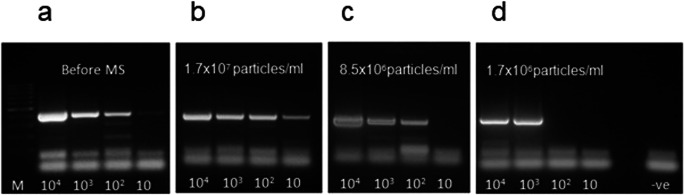
MAP cell capture from spiked broth suspensions (10 to 10^4^ cells/ml) processed through PhMS using decreasing amounts of D29-coated paramagnetic beads assessed by IS900 PCR. Results are for DNA extracted by boiling at 99 °C for 25 min from samples **a** before magnetic separation (before MS), and immediately after PhMS using: **b** 10 μl of coated beads prepared using 10 mg BcMag^TM^ Tosylactivated beads (final concentration 1.7 × 10^7^ beads/ml of test sample). **c** 5 μl of coated beads prepared using 10 mg BcMag^TM^ Tosylactivated beads (final concentration 8.5 × 10^6^ beads/ml of test sample), and **d** 1 μl of coated beads prepared using 10 mg BcMag^TM^ Tosylactivated beads (final concentration 1.7 × 10^6^ beads/ml of test sample). M: TrackIt 100 bp DNA Ladder (Thermo Fisher Scientific), -ve: negative control

A high level of MAP recovery was observed when PhMS using the phage-coated beads was applied to broth spiked at four levels with MAP. Comparison of IS900 PCR results for samples tested before and after PhMS indicated successful recovery of MAP from all the spiking levels when D29 phage-coated BcMag^TM^ Tosylactivated beads not previously blocked with BSA were employed. As shown in Fig. 1, the analytical sensitivity of the novel phage-magnetic separation was visually similar to that of samples subjected to PMS. In contrast, when phage-coated beads were blocked with BSA, as suggested in the bead manufacturer’s instructions, the capture capability of the phage-coated beads was substantially diminished. The amount of paramagnetic beads used for each PhMS was also found to be a crucial factor to maximize capture sensitivity. PhMS using 10 μl (10 mg) D29 phage-coated beads (equivalent to 1.7 × 10^7^ beads/ml test sample) showed the optimal capture sensitivity and demonstrated successful recovery from all the spiking levels. Use of lower amounts of paramagnetic beads (5 μl and 1 μl/ml sample) was found to adversely affect the detection sensitivity of PhMS (Fig. 2). A similar trend was observed for PhMS carried out using beads prepared with lower phage/MB ratios. Paramagnetic beads coated with 10^10^ PFU/ml bacteriophage (10 PFU/MB) showed the highest MAP detection capability and use of lower phage/MB ratios (5, 3, 1 and 0.1 PFU/MB) adversely impacted sensitivity of the test (data not shown). The lowest detectable number of MAP from spiked broths captured using paramagnetic beads prepared with decreasing PFU/MB ratios was 1–10 MAP/ml for 10 PFU/MB ratio, 10–100 MAP/ml for 5 PFU/MB ratio and 10^2^–10^3^ MAP/ml for 3, 1 and 0.1 PFU/MB ratios, respectively.

### TEM of phage-coated beads

**Fig. 3:**
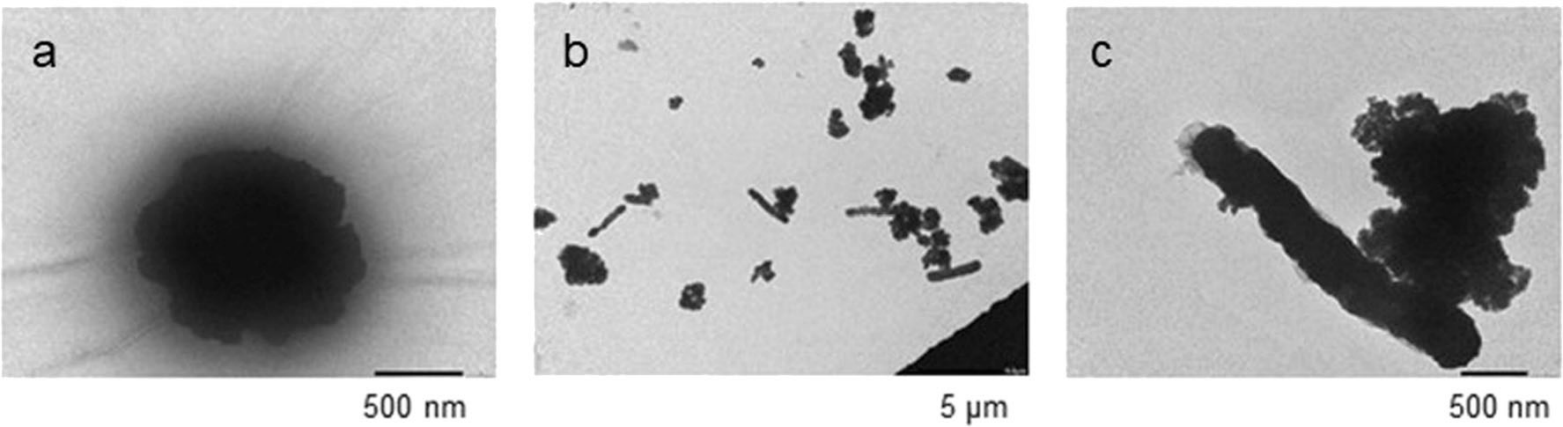
Transmission electron photomicrographs of **a** BcMag Tosylactivated paramagnetic beads coated with D29 phages in ‘tail out’ orientation. **b** Multiple MAP cells captured by phage-coated beads. **c** A single MAP cell attached to single phage-coated bead. Note: the TEM sample preparation procedure has affected the integrity of the beads, so they do not appear as uniform spheres

Figure 3 shows the results of TEM, confirming immobilization of D29 phage particles onto paramagnetic beads in the correct orientation with phage tails pointing outwards from the solid support, and also successful capture of target MAP cells by PhMS.

### Recovery of MAP cells from spiked milk by optimally coated beads

**Fig. 4:**
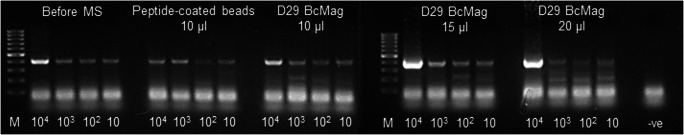
Comparison of the recovery of MAP cells from UHT milk samples spiked at four concentrations (10^4^, 10^3^, 10^2^ and 10 MAP/ml) after MS assessed by IS900 PCR. Results are for DNA extracted by boiling at 99 °C for 25 min from samples before MS and immediately after PMS using 10 μl of peptide-coated MyOne Tosylactivated Dynabeads and PhMS using 10, 15 and 20 μl (1.7 × 10^7^, 2.2 × 10^7^ and 3.4 × 10^7^ beads/ml of sample, respectively) of the optimally coated D29 phage-coated BcMag Tosylactivated beads. M: TrackIt 100 bp DNA Ladder (Thermo Fisher Scientific), -ve: negative control

Once the optimal conditions for coating phages onto paramagnetic beads had been established, an experiment was carried to assess recovery of MAP from UHT milk samples spiked at four levels with MAP using optimally coated D29 phage beads. Three different quantities of D29 phage-coated beads (10 μl, 15 μl and 20 μl) were tested to identify the quantity required per sample for optimal recovery of MAP cells from milk. As shown in Fig. 4, similar detection sensitivity was observed for the three different quantities of D29 phage-coated beads compared. MAP was successfully detected from all the spiking levels in all cases. However, the appearance of PCR bands for samples subjected to PhMS using 15 μl of phage-coated beads indicated a slightly higher intensity in samples spiked at the highest level followed by a more gradual reduction of the intensity of the PCR bands in all the other samples at lower MAP spiking levels. The appearance of the PCR bands suggested greater recovery of MAP cells when 15 μl of phage-coated beads, rather than 10 μl, was used for PhMS. Use of a higher quantity of beads (20 μl/ml of resuspended milk pellet) did not result on any apparent improvement in MAP capture capability.

### Optimization of post-PhMS conditions to maximize release of MAP DNA

**Fig. 5:**
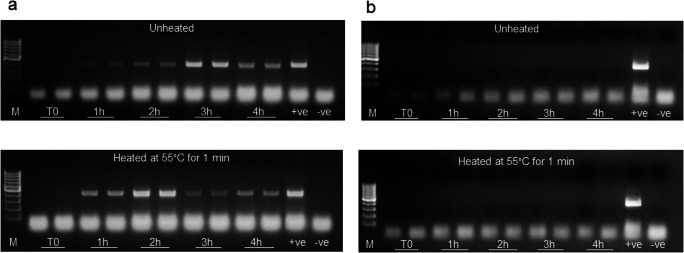
Results of IS900 PCR after PhMS applied to broth suspensions spiked at 10^4^ cells/ml with **a** viable MAP cells and **b** MAP cells inactivated by a 10 kGy dose of gamma radiation. After PhMS, all samples were incubated at 37 °C for increasing incubation times before being subjected to IS900 PCR applied without (unheated samples) and with prior heating at 55 °C from 1 min (heated samples). M: TrackIt 100 bp DNA Ladder (Thermo Fisher Scientific), +ve: positive MAP DNA control, -ve: negative water only control

Our aim was to identify the steps required post-PhMS in order to maximize the quantity of DNA released from viable MAP cells incubated over time with D29 phage-coated beads. The intensity of PCR bands was subjectively assessed at the end of each hour of incubation post-PhMS, with and without a brief heat shock at 55 °C for 1 min. Results indicated an increasing quantity of DNA detected over time, as was expected, with maximum quantity of DNA observed after 2 and 3 h of incubation at 37 °C in the presence and absence of the heat shock treatment, respectively (Fig. 5a). After the 2- and 3-h time points, an apparent reduction in PCR signal was observed, which may indicate adverse effect of phage restriction enzymes on MAP DNA, potentially reducing integrity of DNA molecules suitable for PCR amplification. The brief heat shock clearly had a positive effect on the release of DNA, presumably because phage-weakened MAP cell walls lysed more easily, and the maximum quantity of DNA was released after 2 h rather than 3 h. This suggested that the addition of this brief heat shock had the potential to reduce the overall time of the PhMS-qPCR assay. No PCR bands were ever observed from samples processed immediately after PhMS (T0), confirming that the mild heating step alone was not sufficient to lyse MAP cells. The DNA detected at the end of the test only came from viable MAP cells infected with D29 bacteriophages that had burst as a result of the lytic cycle.

### Confirmation that the PhMS-PCR assay only detects viable MAP

In order to assess the specificity of the new test for viable MAP cells, the above experiment was repeated with broth samples spiked (10^3^–10^4^ MAP/ml) with a radiation-killed MAP cell suspension. DNA release was monitored over time during incubation at 37 °C following PhMS and with and without a brief heat shock at 55 °C for 1 min. No PCR bands were observed for either non-heated or heat-shocked samples (Fig. 5b). Thus, it was demonstrated that D29 bacteriophage can only complete its lytic cycle within viable MAP cells, and that DNA detected at the end of the PhMS-qPCR assay is a reliable indicator of viable MAP being present in the original sample.

### Combine optimised phage-mediated MAP capture and lysis with quantitative qPCR

**Fig. 6:**
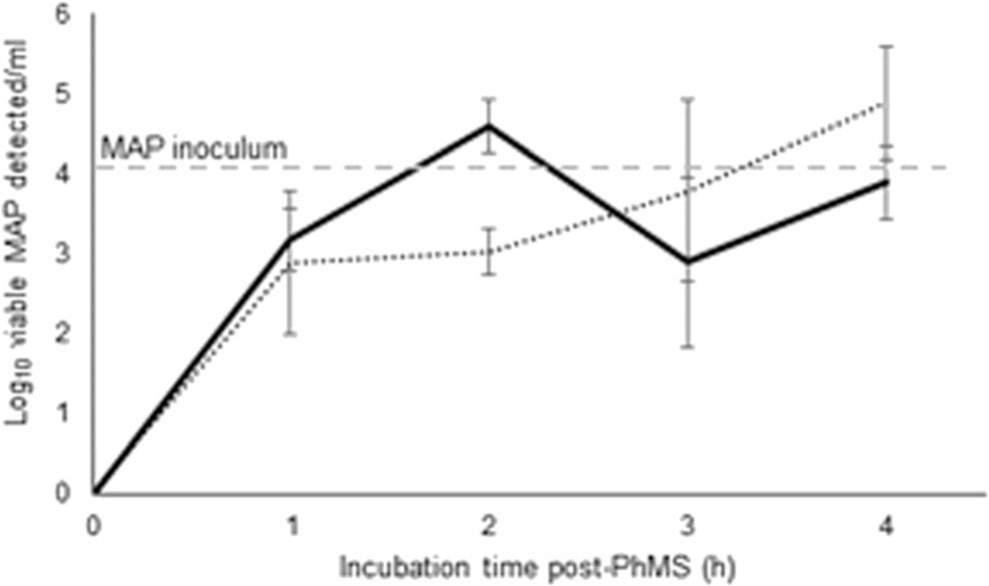
Numbers of viable MAP detected by IS900 TaqMan qPCR in broth suspensions spiked with MAP at 10^4^ cells/ml (indicated by dashed line) following PhMS and incubation of samples at 37 °C for 1–4 h, with (solid line) and without (dotted line) a heat treatment at 55 °C for 1 min. Results are mean log_10_ MAP count/ml ± standard deviation of three MAP strains tested in separate experiments

**Fig. 7:**
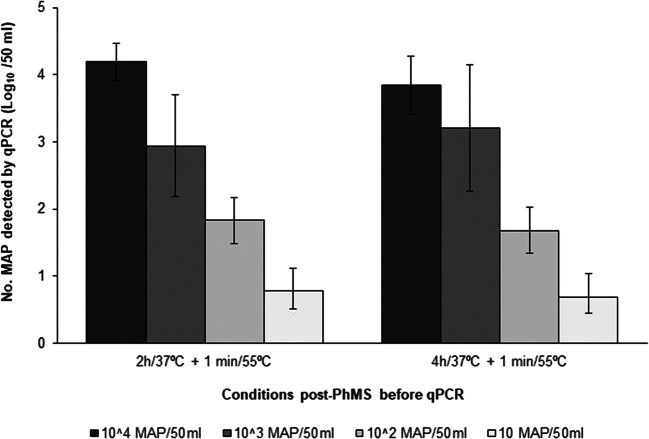
Numbers of MAP detected by PhMS-qPCR assay in UHT milk samples spiked at four levels (10^4^, 10^3^, 10^2^ and 10 cells/50 ml) with three laboratory-grown MAP strains. Before IS900 TaqMan qPCR, samples were processed through PhMS followed by incubation for 2 h or 4 h at 37 °C and a mild heat treatment at 55 °C for 1 min. Results represent mean counts of viable MAP ± standard deviation for three MAP strains tested in three separate experiments

Three different qPCR methods were evaluated to assess which should be combined with the novel phagomagnetic separation method. Application of IS900 TaqMan qPCR provided clearer information about the quantity of DNA from detected MAP cells and showed superior detection rates than the other two qPCR protocols applied in parallel. The novel PhMS method was initially employed in combination with IS900 TaqMan qPCR to test recovery of MAP DNA from broth suspensions spiked at high level (10^4^ MAP/ml) with three different lab-grown MAP strains originally isolated from milk. Results indicated close to 100% MAP recovery after 3 h of incubation at 37 °C post-PhMS (Fig. 6). Slightly higher numbers of MAP were consistently detected from all the three MAP strains 1 h earlier (after 2 h incubation instead of 3 h at 37 °C) if samples were briefly heat-shocked at 55 °C for 1 min at the end of the 2-h incubation period, to lyse MAP cells already weakened by phage lytic action. A second round of experiments was carried out to assess the analytical sensitivity of the test to detect MAP in 50 ml UHT milk samples spiked at four levels (10 to 10^4^ MAP/50 ml). Two versions of the test involving incubation post-PhMS at 37 °C for 2 h (quick version) and 4 h (long version), followed in both cases by a mild heating at 55 °C for 1 min and IS900 TaqMan qPCR, were tested in parallel for comparison. Results of experiments carried out with three MAP strains demonstrated successful detection of 10 MAP cells/50 ml of milk in both cases, and no significant difference in detection sensitivity (*P* value = 0.940319, paired *t* test) between the two versions of the test (Fig. 7). A lower level of detection was observed for the other two qPCR protocols applied in parallel to IS900 TaqMan qPCR. A higher detection limit (> 100 MAP/ml broth or /50 ml milk) was achieved with the commercially available Techne qPCR kit (data not shown), possibly due to the lower number of copies of the f57 gene in MAP genome compared with IS900. No DNA amplification was observed when IS900 SYBR Green was used in combination with PhMS (data not shown), possibly due to OADC or CaCl2 in the bead suspension buffer inhibiting the SYBR biochemical reaction.

### Detection sensitivity of the new PhMS-qPCR test

Blind testing of artificially contaminated UHT milk samples was carried out to determine detection sensitivity and specificity of the test for MAP. A total of 25 UHT milk samples, including 20 samples spiked with MAP at four different levels (five spiked with 10^3^–10^4^ MAP/50 ml; five spiked with 10^2^–10^3^ MAP/50 ml; five spiked with 10–10^2^ MAP/50 ml and five spiked with 1–10 MAP/50 ml) and five non-spiked milk samples, were tested. MAP was successfully detected in all five samples spiked at 10^3^–10^4^ MAP/50 ml and 10^2^–10^3^MAP/50 ml, but in only 3 out of 5 samples spiked with both 10–10^2^ MAP/50 ml and 1–10 MAP/50 ml. No viable MAP cells were detected in any of the five non-spiked UHT milk samples. Estimated limit of detection 50% (LOD_50%_) was calculated to be 10.004 (95% CI 1.20–82.83) MAP cells/50 ml using the online Excel LOD_50%_ calculator.

### PhMS-qPCR testing of BTM samples

Analysis of BTM samples confirmed that the PhMS-qPCR assay was a sensitive test for viable MAP. Forty-nine (49%) of the 100 BTM tested PhMS-qPCR positive for viable MAP, with the number of viable MAP detected ranging from 3 and 126 MAP/50 ml. The vast majority (71%) of the MAP-positive BTM samples contained between 1 and 10 MAP/50 ml milk. The limited volumes of BTM available for testing meant that culture could not be carried out in parallel with the phage-based assay in order to confirm the presence of viable MAP in PhMS-qPCR-positive milk samples.

### Evaluation of the shelf-life D29 phage-coated beads

**Fig. 8:**
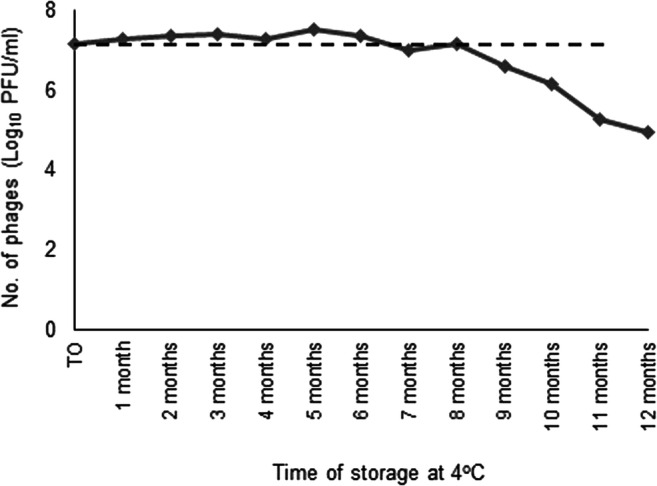
Stability of D29 phage-coated BcMag Tosylactivated paramagnetic beads during refrigerated storage at 4 °C for 12 months assessed by plaque assay

Of the four different storage buffers tested (PBS pH 7.4, 50 mM Tris-HCl pH 9, glycerol 50% and 7H9-OADC-2 mM CaCl_2_ broth), only the D29 phage-coated beads stored in 7H9-OADC-2 mM CaCl_2_ broth maintained their MAP capture capability and demonstrated recovery of MAP cells from all four spiking levels tested, even after 6 months of storage at 4 °C. The bead stocks resuspended in the three other storage buffers showed visible drop off in capture capability after a couple of months of storage (data not shown). A new stock of D29 phage-coated beads was prepared and stored in 7H9-OADC-2 mM CaCl_2_ at 4 °C and tested monthly for 12 months through the plaque assay. Phage numbers remained constant for 8 months at 4 °C, but then progressively declined over the next 4 months of storage by 2 log_10_ (Fig. 8).

## Discussion

Bacteriophages can be used in various ways for the detection of pathogens (Schmelcher and Loessner 2014; Foddai and Grant 2020). The most common lytic phage-based test is the phage amplification assay, or simply the plaque assay. The original FASTPlaqueTB assay for *Mycobacterium tuberculosis* and the PMS-phage assay for MAP are examples of phage amplification assays. Both tests rely upon phage-infected mycobacterial cells being plated with molten agar and fast-growing *M. smegmatis* before the lytic cycle of the phage is completed. When phage-infected *M. tuberculosis* or MAP cells burst in situ within the agar, they release progeny phages which create a plaque (zone of clearing) around the initiator *M. tuberculosis* or MAP cell (or clump) by repeatedly infecting and bursting *M. smegmatis* cells in the surrounding lawn. Unfortunately, due to the fact that the D29 phage can infect a range of *Mycobacterium* spp. (Rybniker et al. 2006) in addition to *M. tuberculosis*, MAP and *M. smegmatis*, the observation of plaques is not definitive proof of the presence of viable *M. tuberculosis* or MAP in a sample. DNA must be harvested from some plaques and target pathogen-specific PCR performed to confirm this. In contrast, for the new PhMS-qPCR assay reported here, the lytic D29 phage was immobilized on Tosylactivated paramagnetic beads to be used for phage-mediated capture of MAP cells, and once captured phage infection of MAP cells would have been initiated also.

To our knowledge, the only other published PhMS assay is a method using phage P22 coated onto Tosylactivated M-280 Dynabeads to capture *Salmonella* Typhimurium cells, before detection in an immunoassay format using specific anti-*Salmonella* antibodies conjugated to horseradish peroxidase as an optical reporter (Laube et al. 2014). These authors did not choose to take advantage of the fact that phage-captured *Salmonella* cells would subsequently be lysed due to P22 phage action. In contrast, for our novel assay, we did choose to wait for cell lysis to occur naturally, so that a test for viable MAP would be achieved. Only viable MAP cells will support amplification of the D29 phage internally resulting in subsequent lysis. Thus, for our test, PhMS of MAP from milk was followed by an incubation period at 37 °C to allow amplification of D29 phages within the infected MAP cells, to the point that the cells burst from within by action of phage endolysins and host cell DNA was released and became available for qPCR confirmation and quantitation of MAP. We found that inclusion of a brief heat shock (55 °C for 1 min) at the end of incubation, mimicking the temperature of molten agar during plating in the PMS-phage assay, aided the earlier, maximal release of DNA from phage-weakened MAP cells (Figs. 5 and 6). Table 1 provides a summary of the key differences between our previous PMS-phage assay and the new PhMS-qPCR assay.

**Table 1:** Summary of the differences between PMS-phage and PhMS-qPCR assays

PMS-phage assay (Foddai and Grant 2017)	PhMS-qPCR assay (this study)
Peptides used to capture MAP cells and phage assay carried out subsequently	Phages used to both capture and infect MAP cells simultaneously
FAS treatment needed to inactivate exogenous seed phage	Not required; presence of phages on magnetic beads is irrelevant to test outcome
Plating of sample with *M. smegmatis* and molten 7H9 agar required	Not required; endpoint of test is no longer plaque formation but qPCR
Brief heat shock when sample is plated with tempered (55 °C) molten agar	Brief heat shock (55 °C/1 min) applied to aid earlier MAP cell lysis
MAP cells burst within agar necessitating extraction of DNA from plaques before PCR	MAP cells burst to release DNA into 50 μl volume, so no other DNA extraction required
Plaques after overnight incubation can be counted, but confirmation they are due to MAP requires plaque PCR	IS900 TaqMan qPCR permits specific detection and quantitation of MAP
DNA from only 10 plaques is typically harvested, irrespective of plaque number observed	Viable MAP cells in entire sample contribute towards template DNA available for qPCR
Total assay time is 24–48 h	Total assay time is ~ 7 h

In the course of test development, a number of things needed to be confirmed/optimised in order to maximize subsequent MAP cell capture, including confirming correct phage orientation on the paramagnetic bead surface by TEM, bead to phage ratio to use during bead coating, number of phage-coated beads to add per PhMS reaction and optimal storage buffer for coated beads. The results presented in Figs. 1, 2, 3 and 4 should clearly demonstrate our decision-making in relation to these parameters, on the basis of conventional IS900 PCR testing immediately after PhMS. We had previously used this subjective evaluation approach to successfully optimise MS methods for MAP (Foddai et al. 2010b; O’Brien et al. 2016) and *Mycobacterium bovis* (Stewart et al. 2012). Our objective was to achieve similar or better MAP capture capability and detection sensitivity with the D29 phage-coated BcMag Tosylactivated beads as we had previously with biotinylated peptide-coated Tosylactivated Dynabeads, and we have done this (Figs. 1 and 4). We also demonstrated that only viable MAP cells can support phage amplification, and consequently be lysed by phage action during the incubation period following PhMS to contribute DNA for qPCR purposes (Fig. 5). Furthermore, we showed that the brief heat shock at 55 °C for 1 min did not contribute enough heat to lyse non-viable MAP cells in a sample (Fig. 5b), which is a vitally important consideration in terms of specificity of the PhMS-qPCR assay for viable MAP only.

Real-time qPCR was selected as the endpoint detection method after PhMS because it provides rapid and quantitative results. We evaluated three different qPCR endpoint detection options to combine with PhMS—two published IS900 qPCR assays (one SYBR green-based (Bull et al. 2014), the other TaqMan probe-based (Sidoti et al. 2011)) and a commercially available qPCR kit for MAP targeting f57 rather than IS900. All three methods had quantitation potential so long as a standard MAP DNA curve was run alongside samples. The TaqMan qPCR and the commercial qPCR kit detected MAP after PhMS, whereas the SYBR green qPCR assay did not yield any positive results. The difference in detection sensitivity of the commercial MAP f57 qPCR kit compared with the TaqMan IS900 qPCR would likely be due to the lower copy number of the f57 target than the IS900 target in MAP cells. Further investigation of why the SYBR green assay failed revealed that the OADC component of the 7H9-OADC-2 mM CaCl_2_ broth used to resuspend the magnetic beads after PhMS, which was still present at DNA template stage, caused inhibition of the SYBR green PCR amplification. These results demonstrate that potential users of the phage-coated beads could combine them with whichever MAP-specific qPCR they are familiar with in their laboratory.

In order to demonstrate that the new PhMS-qPCR assay was capable of detecting viable MAP in naturally contaminated milk samples, and also to show a potential application of the new PhMS-qPCR test (i.e. milk surveillance), 100 BTM samples kindly provided by a local dairy cooperative were tested. MAP counts indicated by the PhMS-qPCR results were in line with previous reports of levels of viable MAP in BTM samples elsewhere (Foddai et al. 2011; Foddai and Grant 2017; Slana et al. 2008, 2009), although the detection of viable MAP in 49% of these BTM samples from Northern Ireland dairy herds was a little surprising and higher than anticipated. It was not possible to culture the 100 BTM in parallel with PhMS-qPCR due to the low volumes of milk recording samples available to us. Had this been possible, the validity of a PhMS-qPCR-positive result as an indication of the presence of viable MAP could have been verified. More extensive PhMS-qPCR testing and culture of BTM and individual milks in comparison with milk-ELISA testing (the test routinely used to screen dairy herds within JD control programmes in several endemically infected countries (Geraghty et al. 2014)) will be reported on in due course.

To conclude, a novel, rapid phage-based PhMS-qPCR test for viable MAP in milk that uses the D29 phage in a different manner to the previous PMS-phage assay was successfully developed and optimised during this study. For naturally infected milk samples, the PhMS-qPCR assay involves PhMS using paramagnetic beads coated with D29 phages, post-PhMS incubation of samples at 37 °C for 4 h (rather than 2 h required for laboratory-grown MAP) followed immediately by a brief heat shock at 55 °C for 1 min, and then MAP-specific IS900 TaqMan qPCR. The whole test takes ~ 7 h, so is potentially a 1-day test. Based on results obtained for BTM, the new PhMS-qPCR assay appears to be a sensitive (LOD_50%_ 10 MAP/50 ml milk), specific, simpler-to-apply and potentially useful phage-based assay for detecting viable MAP in milk.

## Data Availability

Data not included within the manuscript is available upon written request from the corresponding author.
